# Visual tuning in the flashlight fish *Anomalops katoptron* to detect blue, bioluminescent light

**DOI:** 10.1371/journal.pone.0198765

**Published:** 2018-07-11

**Authors:** Melanie D. Mark, Marcel Donner, Dennis Eickelbeck, Jennifer Stepien, Minou Nowrousian, Ulrich Kück, Frank Paris, Jens Hellinger, Stefan Herlitze

**Affiliations:** 1 Department of General Zoology and Neurobiology, Ruhr-University Bochum, Bochum, Germany; 2 Department of General and Molecular Botany, Ruhr-University Bochum, Bochum, Germany; 3 Department of Animal Physiology, Ruhr-University Bochum, Bochum, Germany; University of Sussex, UNITED KINGDOM

## Abstract

Bioluminescence is a fascinating phenomenon and can be found in many different organisms including fish. It has been suggested that bioluminescence is used for example for defense, prey attraction, and for intraspecific communication to attract for example sexual partners. The flashlight fish, *Anomalops katoptron* (*A*. *katoptron*), is a nocturnal fish that produces bioluminescence and lives in shallow waters, which makes it ideal for laboratory studies. In order to understand *A*. *katoptron’s* ability to detect bioluminescent light (480 to 490 nm) at night, we characterized the visual system adaptation of *A*. *katoptron* using phylogenetic, electrophysiological and behavioral studies. We found that the retinae of *A*. *katoptron* contain rods and sparse cones. *A*. *katoptron* retinae express two main visual pigments, rhodopsin (RH1), and to a lesser extent, rhodopsin-like opsin (RH2). Interestingly, recombinant RH1 and RH2 are maximally sensitive to a wavelength of approximately 490 nm light (λ_max_), which correspond to the spectral peak of *in vivo* electroretinogram (ERG) measurements. In addition, behavioral assays revealed that *A*. *katoptron* is attracted by low intensity blue but not red light. Collectively, our results suggest that the *A*. *katoptron* visual system is optimized to detect blue light in the frequency range of its own bioluminescence and residual starlight.

## Introduction

Bioluminescence has evolved in many different organisms such as bacteria, fungi, flies and fishes [1]. It is produced by an oxygen dependent reaction involving a catalyzing enzyme (luciferase) and a complex light-emitting molecule (luciferin) to produce light. Bioluminescence has evolved independently at least 40 times during evolution [2]. The emission of light is normally in the blue/green spectral range (e.g. 440 nm in amphipod shrimps *Scina rassicornis* and *Scina borealis* and 470 nm in the jellyfish *Aequorea victoria*) with some red-shifted exceptions found in crustaceans and tunicates [3].

Bioluminescence often occurs in the marine environment in particular in the deep sea rather than in benthic or shallow waters. Bioluminescent light is often used by fish to detect prey and most likely to communicate [2]. Bioluminescence is found in at least 42 families of fish, where it is produced intrinsically or by bacterial symbionts kept in specialized organs [2]. Since bioluminescence is mainly found in deep-sea fish, it is difficult to study the species-specific function of bioluminescence over long periods of time and under semi-controlled conditions (i.e. in coral reef tanks). However, flashlight fish *A*. *katoptron* is an exception because this species lives in shallow water of coral reefs where they are active at night and hide in reef caves and crevices during the day [4–9]. *A*. *katoptron* is characterized by a bean shaped, torch-like light organ under the eye [7,10]. The light organs are embedded in a suborbital cavity under the eyes and are connected at the anterior edge via a cartilaginous rod like attachment [7]. The suborbital light organ is densely occupied with luminous symbiotic bacteria that produce bluish light at 490 nm in the wavelength spectrum [5,6,11–13]. Emission of light produced by the symbionts is enhanced by a mirror on the back of the light organ [7,9,14]. Furthermore, *A*. *katoptron* produces striking blink patterns during the night [5,7–9], which are used for prey detection [13] and probably for intraspecific communication.

Since *A*. *katoptron* is active during the night and uses bioluminescent light, one can hypothesize that the *A*. *katoptron* visual system has become optimized to detect light in the wavelength range of the bioluminescent signals, namely blue/green light. In this study we investigated the retinal organization and expression of visual pigments in *A*. *katoptron*. We found that *A*. *katoptron* retinae contain rods, very few cones and express mRNAs encoding for two types of visual pigments (i.e. *Rh1* and *Rh2*). In addition, these pigments are maximally activated by blue light, which overlap with the maximal response peaks from ERG recordings and conditioned feeding behavioral assays. Since light-activated G protein coupled receptors such as RH1 are currently used for optogenetic applications [15], the biophysical properties of RH2, i.e. fast kinetics (in comparison to the mammalian RH1) and high light-sensitivity makes RH2 an ideal optogenetic tool. Our results suggest that the *A*. *katoptron* retina is specialized to detect low intensity blue light in the wavelength range of its own bioluminescence. Furthermore, this is one of the first studies to correlate blue light emission with physiological processing and behavioral function.

## Material and methods

### Maintenance of fish

*A*. *katoptron* were imported and purchased from De Jong Marinelife B.V. Spijk, Netherlands. *A*. *katoptron* were caught at the Cebu Islands (Philipines). *A*. *katoptron* were kept in a coral reef tank on a 12 h day and night cycle (for details see [13]). The present study was carried out in accordance with the European Communities Council Directive of 2010 (2010/63/EU) for care of laboratory animals and approved by a local ethics committee (Bezirksamt Arnsberg) and the animal care committee of North Rhine-Westphalia, Germany, based at the LANUV (Landesamt für Umweltschutz, Naturschutz und Verbraucherschutz, Nordrhein-Westfalen, D-45659 Recklinghausen, Germany) (84–02.04.2014.A306). The study was supervised by the animal welfare commissioner of Ruhr-University. All efforts were made to minimize the number of fish used for this study.

### Histology and immunohistochemistry

For the histological analysis of fish retinae Aldehyde-Fuchsin Goldner (AFG) stainings were performed according to the methods of Blüm et al, (1988) [16]. Briefly, four *A*. *katoptron* and three *Carassius auratus* were anaesthetized in 1 g/l MS-222 and fixed in Bouin’s solution (Sigma Aldrich) for 1 week. Tissues were then embedded in Paraplast and sliced in 10 μm sections. Prior to staining of retinal slices, paraffin was removed and sections were rehydrated in distilled water. Retinal slices were then dehydrated, mounted in Euparal, and analyzed on an AxioPlan 2 Microscope System (Zeiss) with a Canon EOS 600D camera.

For the immunohistochemical identification of rods and cones, retinae were dissected and fixed in 4% PFA for 1 h at 4°C and cryoprotected overnight by incubating in 30% (w/v) sucrose in PBS. Samples were embedded in Tissue Tek OCT (optimal cutting temperature) compound and immediately frozen in dry ice. 20 μm cryostat sections were collected and air-dried at room temperature on gelatin-coated slides. Retinal slices were blocked in 20% FBS (fetal bovine serum) and 2% donkey serum in PBS for 1 hr at room temperature and incubated with cone specific marker FITC-PNA (FITC conjugated peanut agglutinin; Sigma), mouse anti-rhodopsin (AbCam, England) and secondary antibodies to donkey anti-mouse Alexa 549 (ThermoFisher Scientific). Images were recorded with a Leica TCS SP5II confocal microscope.

### mRNA isolation, and mRNA sequencing, and identification of opsin transcripts

mRNA was isolated from *A*. *katoptron* retina with Dynabeads mRNA Direct Kit (Ambion, Life Technologies) and quantified with an Agilent RNA 6000 Nano Assay Chip and a 2100 Bioanalyzer (Agilent Technologies). Isolated retinal mRNA was fragmented using a divalent fragmentation buffer in combination with heat, and cDNA was synthesized from the mRNA fragments with random primers. The cDNA was sequenced with an Illumina HiseqTM2000 and assembled with Trinity at BGI (BGI, Hong Kong). The sequenced data produced 184,696,218 total raw reads and 175,873,140 total clean reads (reads were cleaned at BGI using the following criteria to remove reads: reads containing adaptor sequences, reads with more than 5% unknown nucleotides, reads with more than 20% of nucleotides with base quality ≤10). De nova transcriptome assembly was performed at BGI with Trinity [17]. Putative opsin sequences were identified from the assembled transcripts and derived protein sequences using BLAST analyses [18] with zebrafish opsin genes as queries. The gene expression level was calculated by using RPKM (Reads Per kb per Million) [19] method, and the formula used is as follows: RPKM = 10^5^C/NL/10^3^.

### Quantitative reverse transcriptase PCR (qRT-PCR)

mRNA of five *A*. *katoptron* retinae was individually isolated with Dynabeads mRNA Direct Kit (Ambion, Life Technologies) and transcribed with SuperScript III First Strand Synthesis System (Invitrogen, Life Technologies). Serial dilutions from 5^0^ to 5^9^ were prepared from 2 μl of the RT cDNA. Then *Rh1*, *Rh2* and *β actin* (internal loading control) were amplified from cDNAs by qRT-PCR. Duplicate qRT-PCRs were performed for each retina. Relative RNA expression levels were calculated from the band intensities, which were determined by NIH Image J. The following oligonucleotide primers were used for qRT-PCR:

*Rh1*, forward primer: 5`-GAGAGGTGGGTTGTCGTCTG-3`; reverse primer 5`- GGGCAACCAACATACCAGGA -3`;

*Rh2*, forward primer: 5`-TTGGCTGTGGCTGGATTGAT-3`; reverse primer 5`-AGACTGGAACCGTGAAGTGG-3`;

*ß actin*, forward primer: 5`-GCTCGTCGTCGACAACGGCTC-3`; reverse primer 5`-CAAACATGATCTGGGTCATCTTCTC-3`

### Phylogenetic analyses

*Rh1* and *Rh2* sequences were extracted from the transcriptome. Multiple alignments were created in ClustalX, and the same alignment was used for analysis by Neighbor joining methods. Phylogenetic analyses were made with PAUP* version 4.0b10 for Windows (D.L. Swofford, distributed by Sinauer Associates, copyright 2001 Smithsonian Institution) for Neighbor joining. Neighbor joining was performed using 10,000 bootstrap replicates with default parameters (conLevel set to 50, distance measure set to mean character difference). Consensus trees were graphically displayed with FigTree v1.4.2 (http://tree.bio.ed.ac.uk/software/figtree/).

### Generation of plasmid constructs

Reverse transcription of mRNA isolated from *A*. *katoptron* retina and PCR amplification were used to clone *Rh1* and *Rh2*. Oligonucleotide primers were synthesized according to the mRNA sequence information:

*Rh1*, forward primer: 5`-GCGGATCCGGTGCAGGAGACACAGAGGATGCAGA-3`; reverse primer 5`-GCGAATTCATGAATGGCACAGAGGGACCAGAT-3;

*Rh2*, forward primer: 5`-GCGGATCCCCGGACACAGAGGACACTTCTGTCTT-3`; reverse primer: 5`-GGGGAATTCATGGACGTAAATGGAACAGAG-3`. PCR products were cloned into pmCherry-N1 (Clontech Laboratories, Inc.) and the *Rh1* and *Rh2* nucleotide sequences were verified by sequencing. The GenBank accession numbers for *Rh1* and *Rh2* nucleotide sequences are MH460818 and MH460819, respectively. The expression construct *Rh1*-Rat-mCherry (N1) is described in Oh et al., 2010.

### Cell culture

Human embryonic kidney 293 (HEK293) cells were maintained at 37°C in Dulbecco’s modified Eagle’s medium, 4.5 g l^-1^D-glucose, supplemented with 10% fetal bovine serum (Gibco) and 1% penicillin/streptomycin in a humidified incubator under 5% CO2. Growth medium of stable cell lines was supplemented with G418 (5 mg/ml) [20,21]. Stably expressing GIRK1/2 subunits HEK293 cells (kindly provided by Dr. A. Tinker; UCL, London, Great Britain) were transfected with FuGeneHD (Promega) according to the manufactures protocol with *Rh1*-Rat-*mCh* and the newly generated *A*. *katoptron Rh1*-*mCh* and *Rh2*-*mCh* constructs. Transfected cells were incubated for 18–24 h before recordings and performance of cell-based assays.

### Western blot analyses

Western blots from mCh, RH1-mCh and RH2-mCh expressed in tsa201 (HEK cells stably transformed with SV40) cells were performed according to published methods [22]. Briefly cell homogenates were run on a 10% SDS-PAGE, transferred to a PVDF membrane and incubated with the primary antibody rabbit anti-mCh (AbCam, England) and the secondary antibody goat anti-rabbit conjugated to horseradish peroxidase (Novex, ThermoFisher). Membranes were developed with the SuperSignal West Dura Extended Duration Substrate (ThermoFisher Scientific).

### *In vitro* electrophysiology and data analysis

HEK cell recordings: For GIRK channel recordings light-sensitive GPCRs were expressed in HEK293 cells stably expressing GIRK1/2 subunits. Cells were cultured and recorded in dark room conditions after transfection. GIRK-mediated K^+^-currents were measured and analyzed as described previously [23]. The external solution was as follows: 20 mM NaCl, 120 mM KCl, 2 mM CaCl_2_, 1 mM MgCl_2_, 10 mM HEPES-KOH, pH 7.3 (KOH). Patch pipettes (2–5 megaohms) were filled with internal solution: 100 mM potassium aspartate, 40 mM KCl, 5 mM MgATP, 10 mM HEPES-KOH, 5 mM NaCl, 2 mM EGTA, 2 mM MgCl_2_, 0.01 mM GTP, pH 7.3 (KOH). Cells were recorded in external solution containing 1μM 9-cis-retinal (Sigma) unless otherwise stated. Cells were visualized using a trans-illuminated red light (590 nm) or green light filter (480 nm) during experimental manipulations. Whole-cell patch clamp recordings of HEK293 cells were performed with an EPC9 amplifier (HEKA). Currents were digitized at 10 kHz and filtered with the internal 10-kHz three-pole Bessel filter (filter 1) in series with a 2.9-kHz 4-pole Bessel filter (filter 2) of the EPC9 amplifier. Series resistances were partially compensated between 70 and 90%. Cells were incubated in external solution containing 1 μM 9-cis-retinal (Sigma) for 20 min before light stimulation. The PatchMaster software (HEKA) was used for the controls of voltage and data acquisition, and off-line analysis was made with Igor Pro 6.0 software (Wavemetrics).

### Electroretinogram measurements

Experiments were performed in an experimental tank (40 cm length x 20 cm depth x 12 cm height), filled with artificial seawater. Recordings were performed in an electrically grounded Faraday cage. Before the ERG recordings 10 *C*. *auratus* and 10 *A*. *katoptron* were anaesthetized with 0.01 g/l MS-222 and immobilized with an intramuscular injection with pancuronium bromide (0.3 mg kg^-1^). The fishes were dark adapted at least 30 min. Oxygenated seawater containing 0.01 g/l MS-222 was applied to the fish mouth and gills via a 5 mm plastic tube and a peristaltic pump (Aqua medic, Germany). ERGs were recorded through a small incision in the eye surface. Recordings were performed with glass electrodes with 20 μm tip diameter filled with ringer solution. A silver wire reference electrode was attached to the body. The ERG signals were differentially amplified 20000 times with an EXT-02F amplifier system (npi Electronic Instruments for the Life Sciences, Germany). 500 ms light pulses in 10 nm steps ranging from 400 nm to 650 nm with an irradiance of 1.024 to 1.744 photons/m^2^s, respectively (as measured by a Optical Power Sensor, Thor labs) were applied to the eye using a Polychrome V (TILL Photonics, Germany). Spectral responses were plotted against the reciprocal of irradiance for each wavelength. Each fish underwent 5 trials.

### Behavioral experiments

*A*. *katoptron* were kept in a 633 l coral reef tank (135 x 67 x 70 cm) in a 12 h day and night cycle. For details on maintenance of the fish see Hellinger et al., 2017 [13]. One hour after the beginning of the night cycle *A*. *katoptron* were fed with *Artemia spec* and minced salmon. For orientation purposes of the experimenter in the aquarium room, feeding was performed in the presence of high intensity red light delivered by a LED flashlight (2 mW/mm^2^). We used red light, because we initially thought that *A*. *katoptron* would not be able to sense red light. However, this most likely depends on the red light intensity, because high intensity but not low intensity red light activates the recombinant RH1 protein. Thus, the regular feeding during high intensity red light led to a Pavlonian conditioning, i.e. red light (conditioned stimulus) was associated with the beginning of food delivery (unconditioned stimulus). Based on the conditioning responses we analyzed the time how long 1 school of 8 fish spend in the illuminated feeding area using different wavelengths of light (i.e. 460, 480, 530, 630 nm) each delivered with 10% maximal intensity of the monochromatic light source (Polychrom V, Till Photonics). An average of 45 trials/day over 11 consecutive days were performed. For each wavelength and intensity 30 trials were analyzed. Behaving animals were recorded using an infrared (IR) sensitive HD-video camera (Sony HDR-CX 730 6.3 mm CMOS-Sensor, 24.1 megapixel, 6544 x 3680, 30 frames/s sampling rate). The time the fish spend in the illuminated feeding area was calculated using VLC software (Video Lan Client, Version 2.2.1). For illustration see videos in supplemental material.

All values are reported as mean ± SEM. Statistical significance was evaluated with ANOVA: * p < 0.05, ** p < 0.01; *** p < 0.001.

## Results

### The retina of *Anomalops katoptron* consists of rods and sparse cones

**Fig 1:**
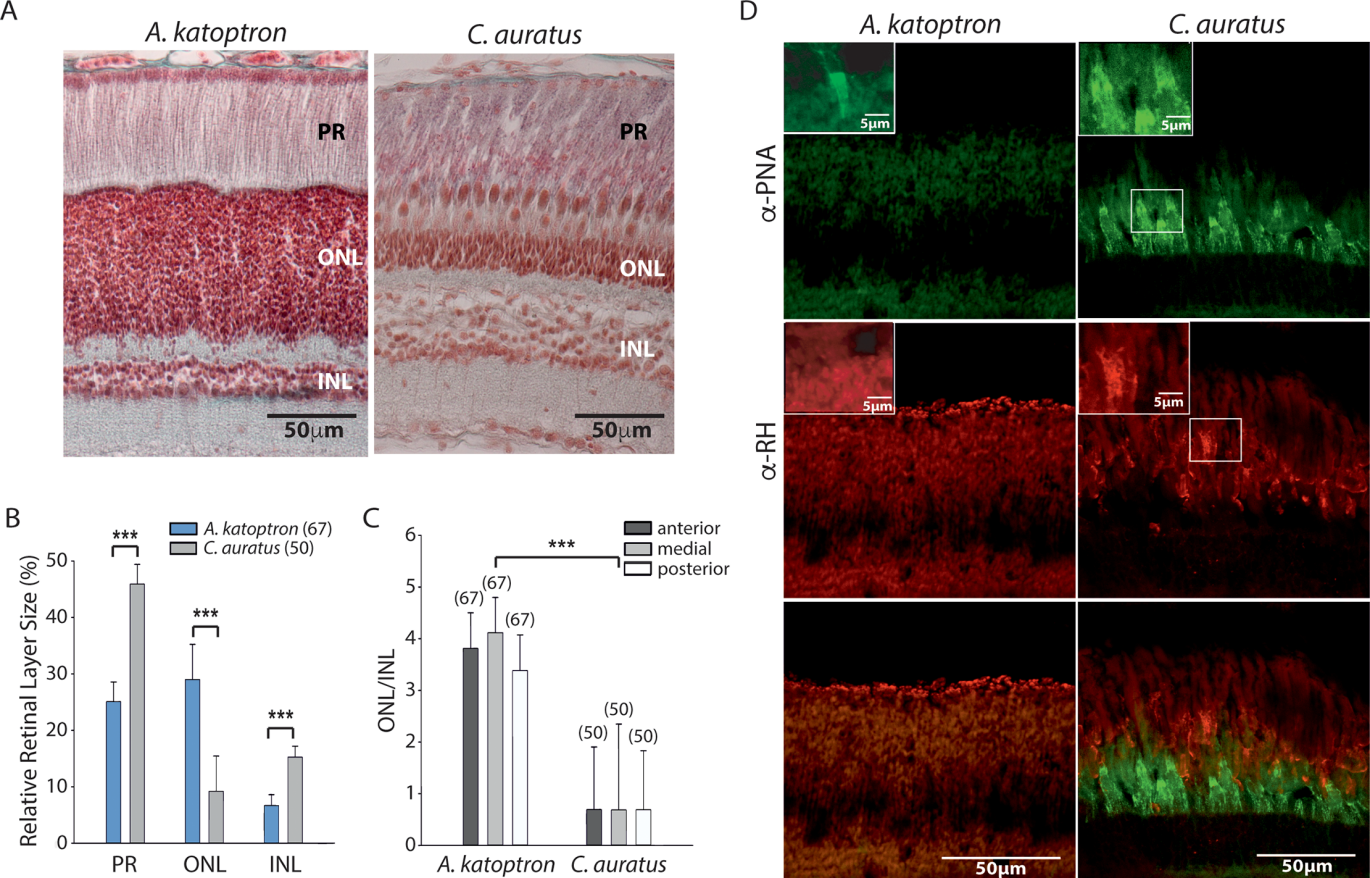
Histological comparison between the retinae of *Anomalops katoptron* and *Carassius auratus*. (A) AFG staining of *A*. *katoptron* (left) and *C*. *auratus* (right) retinae reveal differences in the organization of the different retinal layers. (B) Quantification of the relative size of the different *A*. *katoptron* (blue) and *C*. *auratus* (grey) retina layers (photoreceptor layer (PR), outer nuclear layer (ONL) and inner nuclear (INL) layer), and (C) comparison between the ONL/INL ratio between *A*. *katoptron* (left) and *C*. *auratus* (right). The number of slices analyzed is indicated in parentheses. Statistical significance was evaluated with ANOVA (***p<0.001) (D) Immunohistological analysis of *A*. *katoptron* (left) and *C*. *auratus* (right) retinae. Cones were visualized using FITC-PNA (FITC conjugated peanut agglutinin) in green, and rods were identified by the expression of rhodopsin (RH) in red detected with mouse anti-rhodopsin antibody and donkey anti-mouse Alexa 549 antibody. Rare incidences of cones could be detected in *A*. *katoptron* as shown in the inset. The larger panel from *A*. *katoptron* represents the norm.

To understand the organization of the *A*. *katoptron* visual system we performed a histological analysis of their retina and compared the laminar organization of *A*. *katoptron* to goldfish *Carassius auratus* (*C*. *auratus)* retina. Histological analysis revealed differences in the organization of the *C*. *auratus* retina in comparison to *A*. *katoptron* (Fig 1A). The photoreceptor layer (PR) and inner nuclear layer (INL) in *A*. *katoptron* are relatively smaller, while the outer nuclear layer (ONL) is larger in comparison to the ONL in *C*. *auratus* (Fig 1B). The PR in *A*. *katoptron* contains rods and very few cones (Fig 1A and 1D), while the PR in *C*. *auratus* contains rods and many cones (Fig 1A and 1D). This also becomes obvious when retina slices were fluorescently stained with a cone specific marker (peanut agglutinin (PNA-FITC)) and a rod specific marker (rhodopsin). We could detect cones and rods in the *C*. *auratus* retina, whereas the *A*. *katoptron* retina revealed rods but only very sparse detection of cones by PNA (Fig 1D inset).

### *Anomalops katoptron* expresses the visual pigments RH1 and RH2

**Fig 2:**
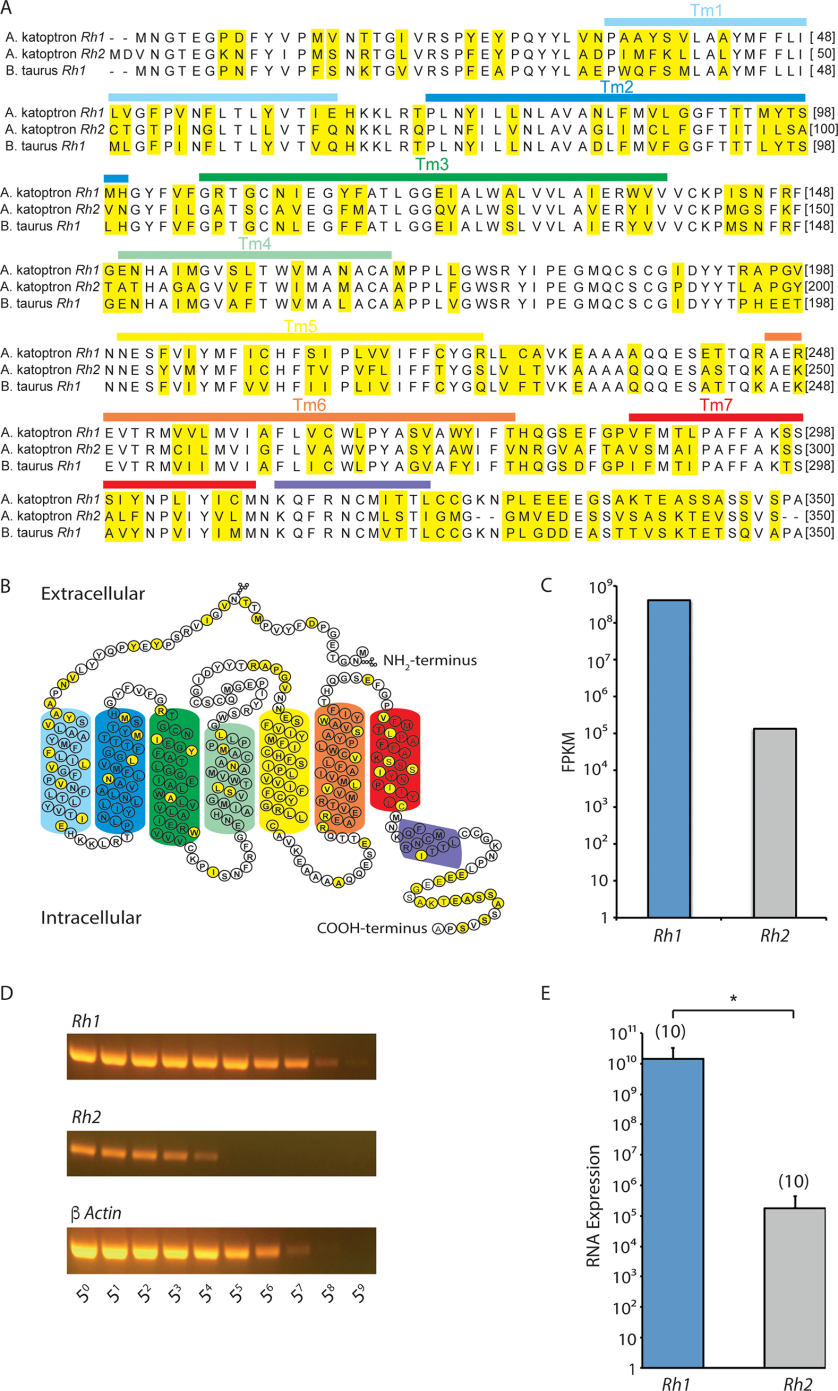
Comparison of the amino acid sequence of *Rh1* and *Rh2* of *Anomalops katoptron* with bovine rhodopsin. **(**A) Amino acid sequence alignment of *A*. *katoptron Rh1*, *Rh2* and bovine *Rh1*. Yellow shows the amino acid substitutions; white shows the conserved amino acids; Tm shows the transmembrane region. (B) 2-D plot of the proposed secondary structure based on the crystal structure of bovine *Rh1*[24]. The amino acid changes in *A*. *katoptron Rh1* are shown in yellow compared to bovine *Rh1*. (C) Comparison of the relative amounts of retinal mRNA of *A*. *katoptron Rh1* and *Rh2* by RNA sequence data. (FPKM, fragments per kilobase million) (D) Example qRT-PCR gels from *Rh1*, *Rh2* and ß actin (internal control) at various dilutions (5^0^ to 5^9^). (E) Relative RNA expression levels of *A*. *katoptron Rh1* and *Rh2* from qRT-PCR. The duplicate qRT-PCRs from 5 different retinae mRNAs were analyzed and indicated in parentheses. Statistical significance was evaluated with ANOVA (*p<0.05).

In order to identify which visual photoreceptors are expressed in the *A*. *katoptron* retina and to compare the nucleotide sequences to other related fish species, we isolated and sequenced the mRNA from the *A*. *katoptron* retina. We identified two types of visual pigments, RH1 and RH2, with high protein sequence similarity to vertebrate rhodopsins (Fig 2). Based on the amino acid sequence we created a 2-D structure of the *A*. *katoptron* RH1 according to the bovine rhodopsin structure (Fig 2B) [24]. The amino acid substitutions are highlighted in yellow. Transcriptomes analysis also revealed that *Rh1* is expressed considerably higher than *Rh2* (Fig 2C). Quantitative reverse transcriptase PCR (qRT-PCR) from 5 different *A*. *katoptron* retinae also confirmed that *Rh1* is expressed substantially more than *Rh2* (Fig 2D and 2E).

**Fig 3:**
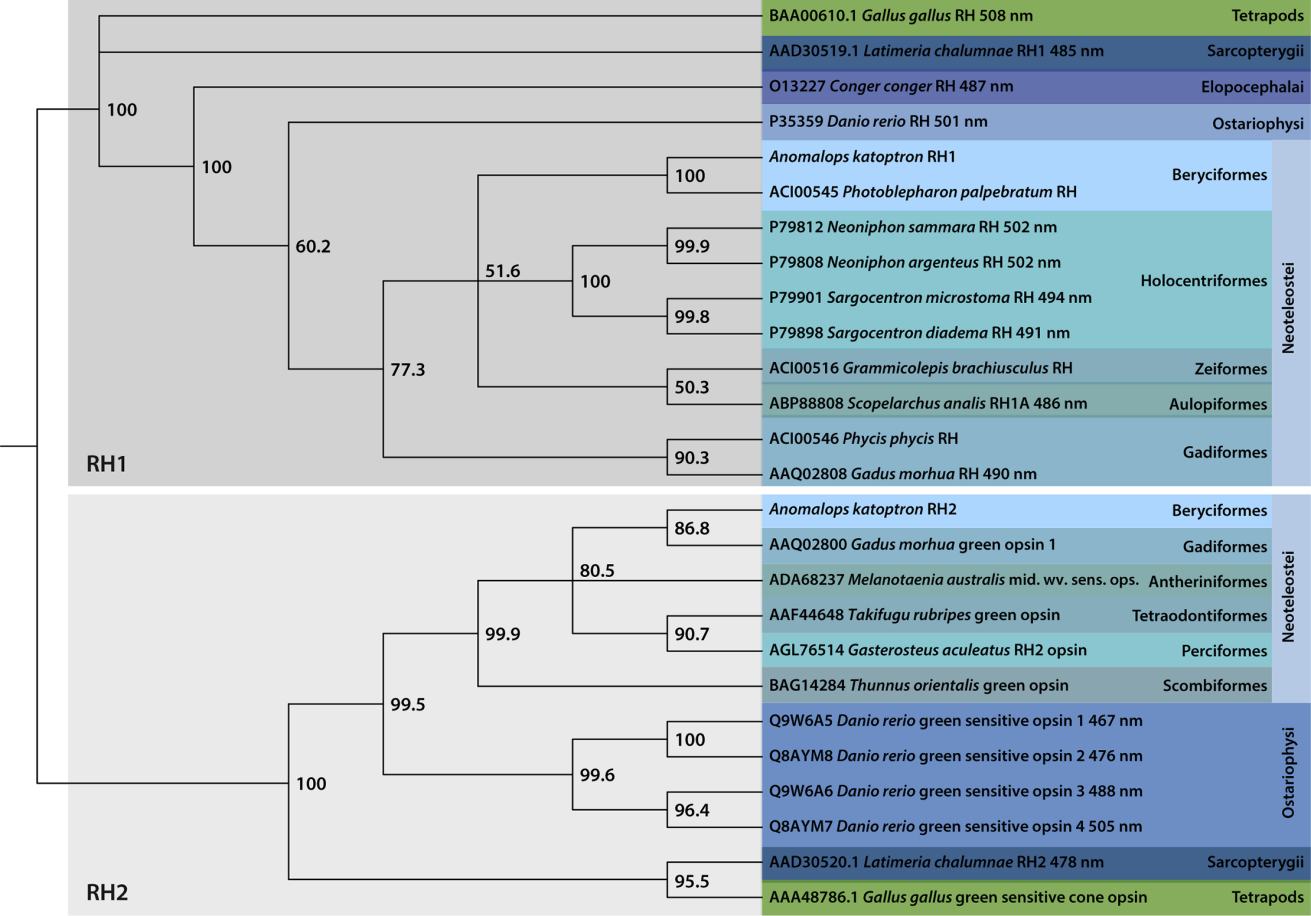
Phylogenetic analysis of rhodopsin proteins from *Anomalps katoptron* and other species. Evolutionary relationship between *A*. *katoptron* RH1 anhd RH2 and other species shown as tree diagram. Phylogenetic trees were generated with Neighbor joining. Bootstrap percentages (10,000 bootstrap replicates) are given at the branches. Classifications given on the right are taken from the NCBI Taxonomy database (http://www.ncbi.nlm.nih.gov/taxonomy) [27].

A phylogenetic analysis with *A*. *katoptron Rh1* and *Rh2* as well as rhodopsin proteins from several fish species and the chicken *Gallus gallus* reveals a clustering of *A*. *katoptron Rh1* and *Rh2* proteins with the different rhodopsins and green sensitive opsins from other species, respectively (Fig 3). The rhodopsin/rhodopsin-like/opsin variants demonstrate absorption spectra with a λ_max_ between 486–502 nm. Within each branch for *Rh1* and *Rh2*, sequences cluster according to species phylogeny. Clustering according to ecological niche (e.g. deep-sea fish) is not observed, which is expected as convergent evolution towards shorter wavelength absorption spectra would most likely involve changes of only a few amino acids, and would therefore not necessarily lead to changes in phylogenetic branching based on full-length protein sequences. For example, *A*. *katoptron Rh1* is most closely related to the partial *Rh1* sequence of the flashlight fish *Photoblepharon palpebratus* (Anomalopidae) and to rhodopsins of squirrelfishes, which live in caves and crevices of coral reefs. In contrast, *A*. *katoptron Rh1* is only distantly related to the deep-sea fishes *G*. *brachiusculus* and *S*. *analis* [25,26]. The *Rh2* sequence of *A*. *katoptron* has the highest similarity to the green opsin of the atlantic cod (*Gadus morhua*; 517 nm).

### Characterization of the action spectra of the visual pigments RH1 and RH2 from *Anomalops katoptron*

**Fig 4:**
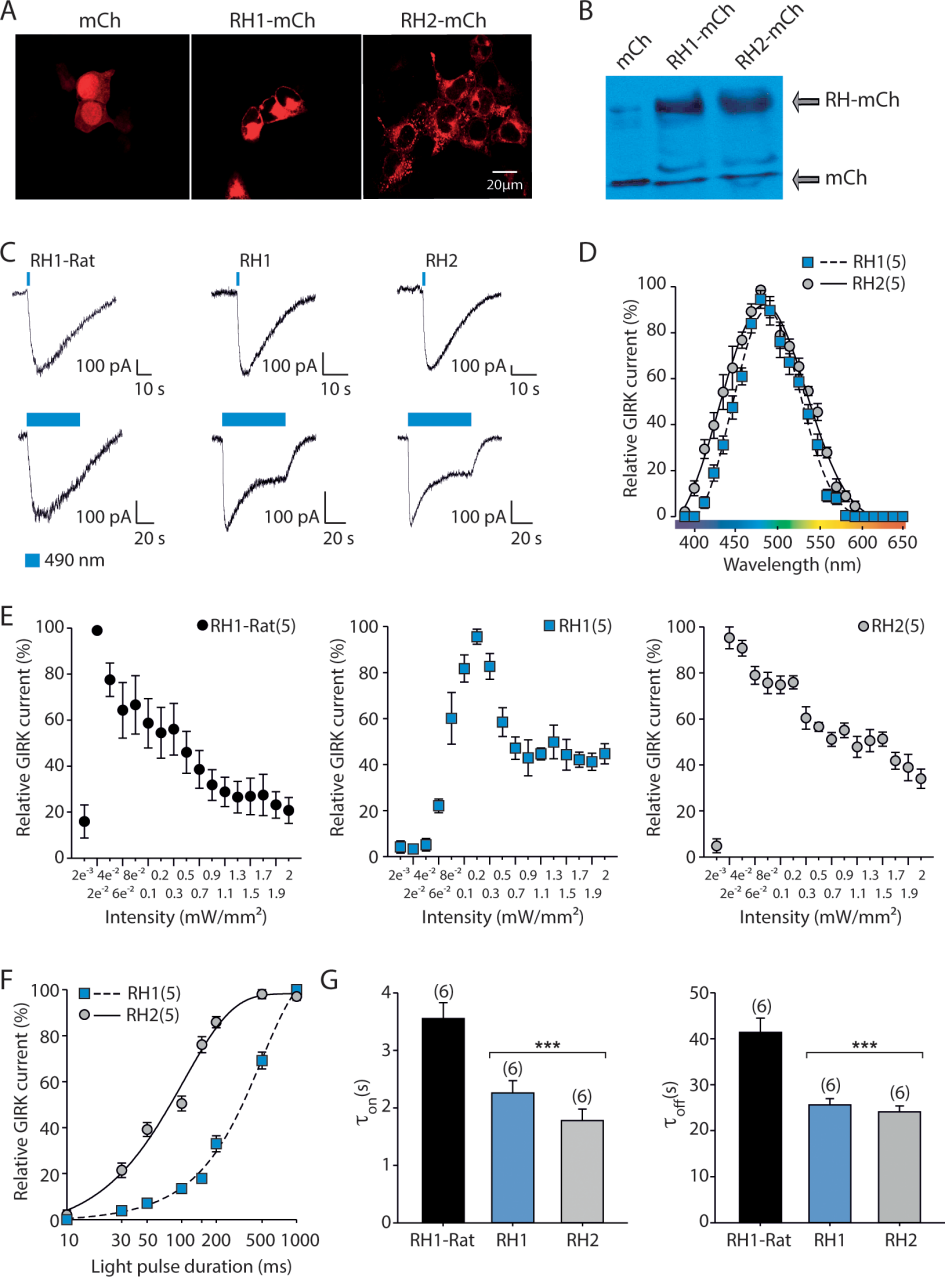
Characterization of the action spectrum and biophysical properties of *Anomalops katoptron* RH1 and RH2. (A) Distribution of mCherry, RH1-mCherry and RH2-mCherry in tsa201 cells. (B) Western blot analysis of tsA201 homogenates expressing mCherry, RH1-mCherry and RH2-mCherry. Protein expression was detected with an antibody against mCherry. (C) Comparison of light-induced GIRK (G protein coupled inward rectifying potassium) currents activated by RH1-Rat, *A*. *katoptron* RH1 and *A*. *katoptron* RH2 using a 1 s or 10 s light pulse of 490 nm (indicated as blue bar). (D) Wavelength dependence of maximal GIRK current activation induced by RH1-Rat, *A*. *katoptron* RH1 and *A*. *katoptron* RH2 using a 1 s light pulse of the indicated pseudorandomized wavelength. (E) Light pulse intensity dependence of maximal GIRK current activation induced by RH1-Rat, *A*. *katoptron* RH1 and *A*. *katoptron* RH2 using a 1 s light pulse of 490 nm with different pseudorandomized light-intensities. (F) Light pulse duration dependence of maximal GIRK current activation induced by *A*. *katoptron* RH1 and *A*. *katoptron* RH2 using a light pulse of 490 nm with increasing time. (G) Activation and deactivation time constants of GIRK currents induced by RH1-Rat, *A*. *katoptron* Rh1 and *A*. *katoptron* RH2 using a light pulse of 470 nm for activation with increasing time. The number of cells analyzed is indicated in parentheses. Statistical significance was evaluated with ANOVA (***p<0.001).

To detect the distribution and expression of RH1 and RH2 in heterologous expression systems both genes were fused to mCherry (mCh). We found that RH1 is targeted to the plasma membrane, while RH2 demonstrates a more clustered distribution in tsa201 cells (Fig 4A). Western blot analyses of RH1-mCh and RH2-mCh lysates revealed an expected protein product of approximately 65 kDa (Fig 4B).

Since the electrophysiological characterization of an action spectra is much more sensitive than biochemical measurements of an absorption spectrum [28,29] and G protein coupled inward rectifying potassium (GIRK) channels are activated via the G_i/o_ pathway in heart, brain and by vertebrate cone and rod opsins, we characterized the wavelength dependent action spectrum of RH1 and RH2 from *A*. *katoptron* using a GIRK channel activated heterologous expression system [21,23,30]. We expressed RH1-mCh and RH2-mCh independently in HEK293 cells stably expressing GIRK1/2 subunits and performed whole-cell voltage clamp recordings following 1 s light pulses of increasing wavelengths from 380 to 650 nm in 10 nm steps. A 1 s light pulse was sufficient to maximally activate RH1 and RH2 mediated GIRK currents (Fig 4C). RH1 and RH2 mediated GIRK currents were maximally induced by 485 to 490 nm (Fig 4D). Thus, the photoreceptor pigments in *A*. *katoptron* are maximally activated by blue light.

We next compared the light sensitivity of the *A*. *katoptron* rhodopsins to rat RH1, because rat RH1 is biophysically very well characterized and is very light-sensitive in comparison for example to microbial opsins [21]. Surprisingly, we found that *A*. *katoptron* RH2 is more light-sensitive than RH1 and its light sensitivity spectrum resembles the rat RH1. A 0.002 mW/mm^2^, 1 s light pulse at 490 nm was sufficient to maximally activate *A*. *katoptron* RH2 and rat RH1, while maximal activation of RH1 required a 0.2 mW/mm^2^ light pulse (Fig 4E). In addition, the half maximal light pulse duration for maximal activation of GIRK currents is around 80 ms for RH2 and 350 ms for RH1 (Fig 4F). The *A*. *katoptron* rhodopsins also differ in their activation and deactivation kinetics when compared to the rat RH1. Both, *A*. *katoptron* RH1 and RH2 have much faster activating (RH1: 2.3 ± 0.2 sec; RH2: 1.8 ± 0.2 sec; rat RH1: 3.6 ± 0.3 sec) and deactivating (RH1: 25.6 ± 1.3 sec; RH2: 24.1 ± 1.3 sec; rat RH1: 41.4 ± 3.1 sec) time constants in comparison to the rat RH1 (Fig 4G). Since the activation and deactivation kinetics of the G protein coupled receptor (GPCR) mediated GIRK currents reflect the intrinsic activation and deactivation kinetics of the light-activated GPCRs in rods and cones [21], these experiments suggest that vision under low intensity light conditions is faster in *A*. *katoptron* than in rats.

### Determining the wavelength dependence on *Anomalops katoptron* retinal signal processing

**Fig 5:**
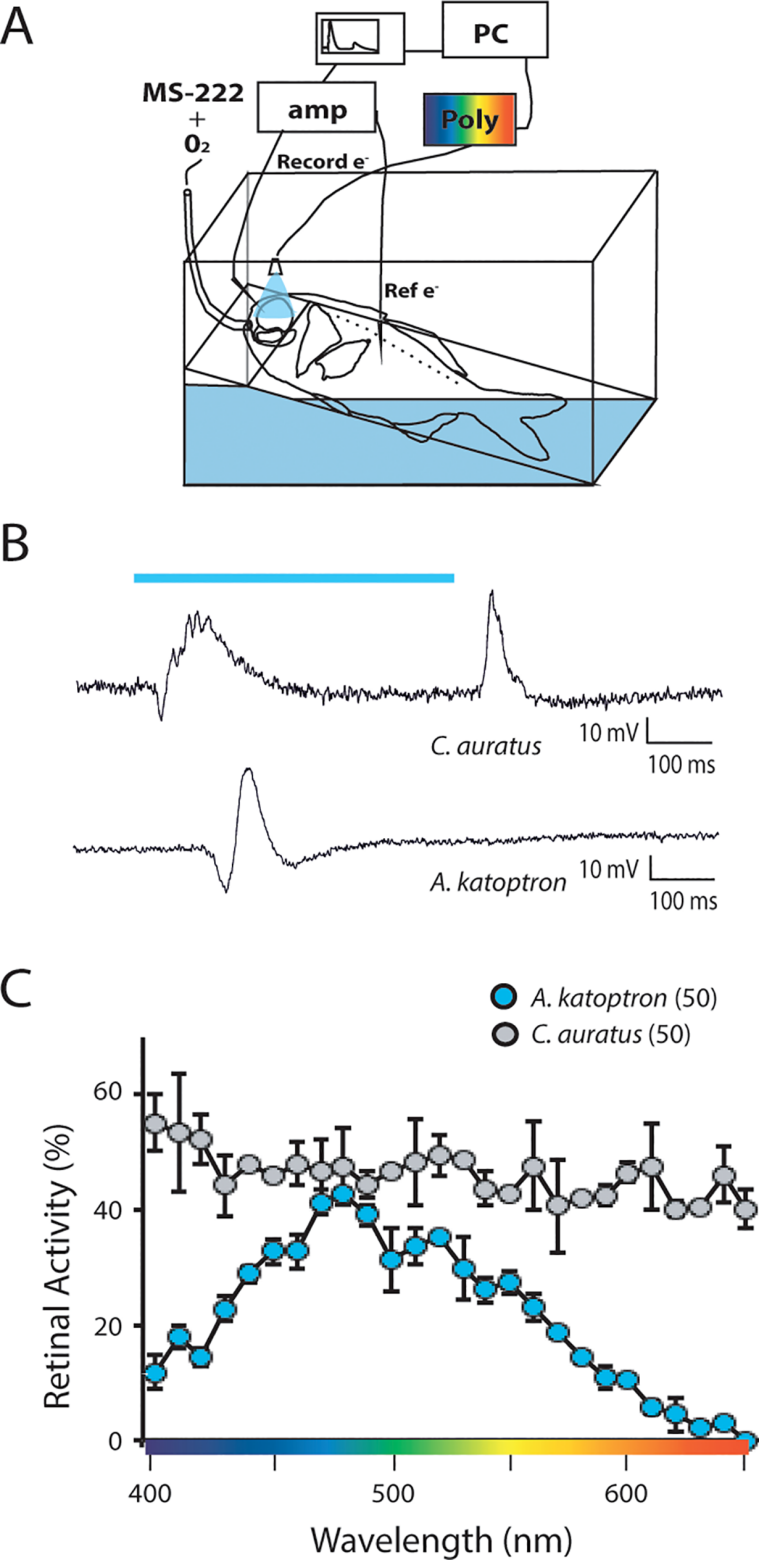
Electroretinogram measurements from *Anomalops katoptron* and *Carassius auratus*. (A) Schematic representation of the experimental set-up to record electroretinograms in fish. Oxygenated (O_2_) seawater containing 0.01 g/l MS-222 was applied to the fish mouth and gills via a 5 mm plastic tube and a peristaltic pump. Light pulses between 400 nm to 650 nm were applied to the retina with a polychromatic light source (Poly). (recording electrode, Record e^-^; reference electrode, Ref e; amplifier, amp; computer, PC) (B-C) Comparison of electroretinogram measurements from *A*. *katoptron* and *C*. *auratus*. (B) Example traces of electroretinograms recorded during a 500 ms, 480 nm light pulse (blue bar) for *C*. *auratus* (top) and *A*. *katoptron* (bottom). (C) Wavelength dependent electroretinogram activities were plotted against the reciprocal of irradiance for each wavelength for *C*. *auratus* (grey) and *A*. *katoptron* (blue) using a 500 ms light pulse for indicated wavelengths. Note that the *C*. *auratus* retinal responses are probably saturating. The number of retinas analyzed is indicated in parentheses.

In order to understand how the *A*. *katoptron* retina perceives and processes light at different wavelengths, we performed ERG measurements under a wide spectrum of light applications to *A*. *katoptron* and *C*. *auratus* retinae (Fig 5). As expected the *C*. *auratus* retinae respond equally well over the entire wavelength spectrum tested (400–650 nm), which were probably attributed to saturating conditions. However, the *A*. *katoptron* retinae were only minimally activated by short (400–420 nm, violet light) and long (580–630 nm, orange/red light) wavelengths of light with a maximal retinal response at 480 nm (blue light; Fig 5C), overlapping with the maximal activation spectrum of RH1 and RH2. Since we detected very sparse cones in the *A*. *katoptron* retina, we did not find the cone related δ-wave responses in the retinogram in comparison to retinogram from C. auratus, which shows this wave after the light pulse [31] (Fig 5B). These results demonstrate that *A katoptron* photoreceptors are spectrally tuned to blue light at wavelengths between 480–510 nm.

### Characterization of the wavelength and intensity dependence on conditioned feeding behavior

**Fig 6:**
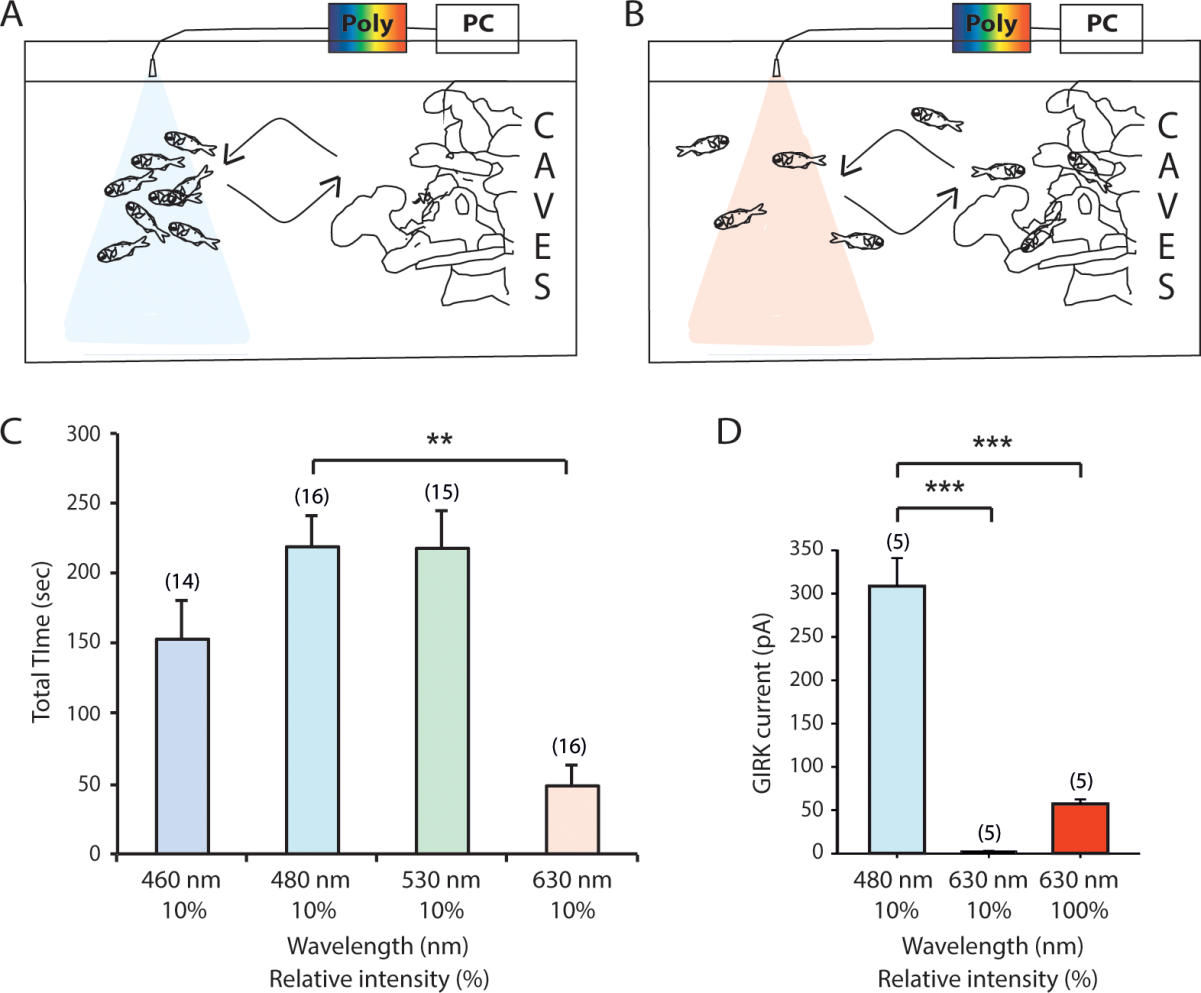
Characterization of the wavelength and intensity dependence on conditioned feeding behavior of *Anomalops katoptron*. (A-B) Schematic representation of the behavioral food conditioning experiment (polychromatic light source, Poly). A school of 8 *A*. *katoptron* fish were trained to recognize food delivery associated with high intensity red light (100% at 630 nm, 2 mW/mm^2^, conditioned stimulus). (A) Low intensity blue light (10% at 480 nm) attracted the fish to the feeding area. (B) Low intensity red light (10% at 630 nm) did not attract the fish to the feeding area. (C) Characterization of the wavelength dependence on conditioned feeding behavior of *A*. *katoptron*. Wavelength dependent feeding behavior of *A*. *katoptron* was measured at 460 nm, 480 nm, 530 nm and 630 nm with 10% light intensities delivered by the polychromatic light source at a given wavelength. (D) Low intensity blue light (10% at 480 nm) but not low intensity red light (10% at 630 nm) activates RH1 mediated GIRK currents. However, high intensity red light (100% at 630 nm) induced RH1 mediated GIRK current, which is about >20% of the current induced by 480 nm (10% intensity). The total number of trials per data point is indicated in parentheses. Statistical significance was evaluated with ANOVA (**p<0.01; ***p<0.001).

To investigate whether the specialization of *A*. *katoptron* retina to blue light detection coincides with behavioral responses to blue light, we trained (conditioned) a school of 8 *A*. *katoptron* fish to recognize the delivery of food associated with light. We initially used a red LED torch (2 mW/mm^2^) on the top of the left side of the aquarium during feeding to illuminate the feeding area for the experimenter (S1 Fig and S1 Video). Surprisingly we found that *A*. *katoptron* associated the occurrence of the red light with feeding. The conditioned feeding response suggests that red light with relatively high intensity (2 mW/mm^2^) induces a photo response in the fish retinae, which is in agreement with data shown in Fig 6D. *A*. *katoptron* RH1 induced GIRK currents were only activated by high intensity (2 mW/mm^2^; 630 nm) but not lower intensity red light (0.2 mW/mm^2^; 630 nm). Thus we tested the wavelength dependence of the conditional light stimulus using low intensity light of different wavelengths. We applied 460 nm, 480 nm, 530 nm and 630 nm wavelengths of low intensity light (0.2 mW/mm^2^) and analyzed if *A*. *katoptron* were attracted to the light beams to receive food (Fig 6A and 6B). *A*. *katoptron* stayed for a long duration in blue-green light (460, 480 and 530 nm; S2 Video) but not in red light (630 nm, Fig 6C and S3 Video) at low light intensities without food delivery. However, *A*. *katoptron* did not stay for a long duration in blue-green light (460, 480 and 530 nm; S1B and S1C Fig and S4 Video) at high light intensities without food delivery. These data suggest that *A*. *katoptron* retinae are specialized to detect low intensity blue/green light, but retinal responses leading to behavioral responses can also be induced by high intensity red light.

## Discussion

### The retinal morphology of the nocturnal fish *Anomalops katoptron*

Nocturnal fish live under low light conditions and therefore need to optimize their visual system to detect low intensity light. Various specializations have been described in nocturnal fish to detect light at night including an increase in eye size, a decrease in the light path, changes in density, distribution, arrangement and connectivity of rods and specialized reflective structures behind the retina (for review see [32]). Accordingly we could mainly detect mRNA for rhodopsin and rhodopsin like genes, but not other cone opsins. The morphology of the *A*. *katoptron* retina may be similar to the retina of the deep sea coelacanth, which also express RH1 and RH2 and compromises about 1–2% of cones [33]. However, the number of cones in *A*. *katoptron* is much lower. In addition, the length ratio between the ONL to INL is larger in *A*. *katoptron* in comparison to *C*. *auratus*, which also suggest that *A*. *katoptron* is specialized to living under low light conditions. Fishes with larger ONL/INL ratio indicate nocturnal species, while fishes with smaller ONL/INL ratio indicate diurnal species [34].

### Tuning the *Anomalops katoptron* visual system to blue light

The visual system of nocturnal and diurnal fish adapted to their photic environment during evolution [33,35]. Daylight vision in fish is dominated by cones and retina of daylight fish expresses up to four different cone pigments to allow color discrimination [35]. On the other hand night vision in fish is dominated by rods and contains rhodopsin to detect low intensity light. In principle visual sensitivity will be adapted to the photic environment by adjusting the spectral tuning of the photoreceptors and/or by varying the number of spectral classes [33][35]. According to the sensitivity hypothesis photoreceptor sensitivity in marine organisms has been adapted between 450–500 nm to absorb as much bioluminescent light or residual daylight from the water surface as possible [35]. In the nocturnal *A*. *katoptron* we found the expression of rod rhodopsin RH1 and the closely related RH2, but we could not detect other visual cone pigments. The expression of only RH1 and RH2 agrees with the very sparse number of cones, predominantly rod containing retina morphology and the absence of cone responses in the electroretinograms (Figs 1A and 5B). Retinal mRNA levels of *Rh1* are much higher than *Rh2* (according to values of quantitative PCR 4500 fold higher), suggesting that the visual system of *A*. *katoptron* is dominated by RH1. RH1 and RH2 from *A*. *katoptron* have a maximal light sensitivity between 485–490 nm, which corresponds to the wavelength of the bioluminescent light of other organisms and in particular to the wavelength of the bioluminescent light emitted by the *A*. *katoptron* light organ [3,13]. This also correlates with the spectral sensitivity from the *A*. *katoptron* ERG recordings, which shows the highest peak activity at 480 nm and a smaller second peak at 510 nm. It has been suggested that the differences in the action spectrum between RH1 and RH2 in coelacanths could contribute to a rod/cone color vision within the blue light spectrum [36]. However, cones are very sparse in the retina of *A*. *katoptron* and therefore color discrimination might not be feasible. Surprisingly, RH2 has a greater light sensitivity than RH1, suggesting a critical role of RH2 under very low intensity light conditions. RH2 is normally expressed in cones and the low number of cones detected in the retina of *A*. *katoptron* may correlate with the relative low mRNA levels of *Rh2* in comparison to *Rh1*. However, cone opsins have also been found in a specialized form of rods in amphibians [37]. Since RH2 has much faster activation kinetics and is more sensitive to light than RH1 in *A*. *katoptron* (see Fig 4E), one can speculate that RH2 expressed in cones sparsely distributed throughout the retina might be used for fast detection of very low intensity blue light in for example escape behavior.

### The evolutionary relationships of *Anomalops katoptron* visual pigments

Vertebrate visual pigments are grouped into 5 different families [38]. These groups include the mid and long wavelength sensitive opsin pigments (M/LWS), the short wavelength sensitive opsin pigments (SWS1 and SWS2), and the rhodopsin pigments (RH1 and RH2). We could detect in our study mRNA for *Rh1* and *Rh2* in the retinae from *A*. *katoptron*. *Rh1* and *Rh2* have been proposed to arise from gene duplication and are the closest relatives within the opsin family [33,39]. RH1 is maximally activated around 500 nm and RH2 around 470–510 nm [40] suggesting that the expression of these two opsins allow for detection of blue/green light. In order to predict and compare the structure function relationships for retinal binding and wavelength specificity of the different opsin subfamilies, a conserved sequence of 35 amino acids forming the retinal binding pocket was deduced [40]. Among these residues RH1 reveals a D83N in transmembrane domain 2 (TM2) and a A292S (bovine; A299S *A*. *katoptron*) substitution in TM7 suggesting a 6 nm and 10 nm blue shift in comparison to rod rhodopsin[41]. The D83N/A292S substitutions occur very frequently throughout evolution and is also found in coelacanth [42,43]. These substitutions seem to be part of the structural domains for adjusting the wavelength specificity in RH1. RH2 reveals a D83G (TM2) and E122Q (TM3) substitution. E122Q mutation in RH1 results in a 20 nm shift to shorter wavelength, while D83G in RH1 results in a 3 nm shift to longer wavelength [41]. The major structural domain for adjusting the wavelength sensitivity in RH2 seems to be therefore E122Q, which also occurs in zebrafish, medaka, chameleon, gecko, and coelacanth [43]. The λmax of these RH2 variants including the *A*. *katoptron* variant carrying the E122Q mutations are blue shifted in comparison to the ancestral RH2 [43].

Termination/deactivation of the visual light response of rod and cone opsins depend on binding of receptor kinases (GRK1) and arrestin to the intracellular protein domains of the opsin variants [44]. GRK1 binding leads to phosphorylation of serine residues in the C-terminus of the opsin molecule and subsequent binding of arrestin. Various studies suggest that the main phosphorylation sites in the C-terminus of vertebrate RH are Ser334, Ser338 and Ser343. The kinetics of phosphorylation and dephosphorylation are faster at Ser338 and Ser343. Surprisingly, *A*. *katoptron* RH1 contains a S338E (bovine; S340E *A*. *katoptron*) mutation in the C-terminus, while the overall number of Ser/Thr residues within the CT of bovine and A. katoptron RH1 is not changed. This suggests that the deactivation and/or desensitization process might be altered for RH1 from *A*.*katoptron*.

### Evolutionary adaptation to low intensity light conditions in deep water and to bioluminescent light

The adaptation of the visual system of fish to low intensity twilight in the wavelength range between 400–500 nm and detection of light in deep sea environments seems to involve the evolutionary adaptation of light detection using RH1 and RH2 pigments to the dark environment [43]. For example, the Comoran coelacanth, a “living fossil”, lives in a depth of 200 m. At these depths light of only around 480 nm can be perceived. Therefore the coelacanth developed a visual system using RH1 and RH2 with maximal light sensitivities of 478 and 485 nm, respectively [45]. The absorption spectra of RH1 and RH2 in coelacanth are blue shifted in comparison to other species and involve amino acid substitutions in the retinal binding pocket, i.e. RH1 (Q122E/S292A) and RH2 (Q122E/L207M) [36]. *A*. *katoptron* most likely also lives around depths of 200 m during the day, but approaches shallow waters during dark nights. (Note, we have not observed *A*. *katoptron* at the water surface or close to the water surface during bright star, moon or city lights while diving or snorkeling). Light is provided mainly by their bioluminescent light organ, emitting light with a wavelength of around 490 nm, which is slightly red shifted in comparison to day light in the deep sea. Therefore one might suggest that RH1 and RH2 have slightly adapted during evolution to detect blue light in the range between 485 (RH2)– 490 (RH1) nm, since *A*. *katoptron* RH1 does not contain the Q122E mutation and L207M does not occur in *A*. *katoptron* RH2. Adapations to a dark environment are common in numerous deep sea fish species with nearly 750 mesopelagic, 200 bathypelagic and 1000 benthic fish species [46], and many mesopelagic fishes evolved specializations in their visual systems e.g. large pupils lenses or tubular eyes [46]. *Anomalops katoptron* reveal a nearly rod dominated retina. Rod dominated retinae in lanternfishes were assumed as a adaptation to residual light and bioluminescence in the mesopelagial environment [47], and retinal extracts in lanternfish show sensitivity peaks close to 490 nm [48]. We assume that a nearly lack of cones in *A*. *katoptron* is also an adaptation to see intraspecific and potentially interspecific bioluminescent signals. We exclude an adaption to residual moonlight on the reef flat because we never observed *A*. *katoptron* under moonlight in shallow water.

The specialization for blue light detection also becomes obvious in the behavioral experiments. Here *A*. *katoptron* associated low intensity blue light, but not red light with the expectation of food delivery. Exclusive high intensity red light evoked a behavioural response in *A*. *katoptron*. The high intensity response is consistent with the ERG recordings with low retinal activity evoked as a response to stimulation with long wavelength red light. On the other hand high intensity blue and green light evoked an avoidance response in *A*. *katoptron*. The results suggest again that light in the wavelength range between 460–530 nm (i.e. bioluminescent light and light-environment of shallow water reefs at night) can be associated with specific behavior.

In summary, our study describes the histological organization of the nocturnal flashlight fish *A*. *katoptron* retina and identifies two rhodopsin variants, which are evolutionarily optimized for the detection of low intensity blue light in the spectral range of its own bioluminescent light. Biophysical characterization of the wavelength dependent activation and deactivation of *A*. *katoptron* revealed that RH2 is more sensitive to light than RH1 and demonstrates faster activation and deactivation kinetics of light-induced GIRK currents when compared to the rat RH1. One could speculate that the fast activation and deactivation kinetics of RH2 contribute to the detection of fast moving objects during low light intensities. Therefore it will be important to determine the exact localization of RH2 within retinal cells and how RH2 contributes to visual responses in *A*. *katoptron*.

## Supporting information

S1 Fig Characterization of the wavelength and high intensity dependence on conditioned feeding behavior of *Anomalops katoptron*. Schematic representation of the behavioral food conditioning experiment (polychromatic light source, Poly). (A) A school of 8 *A*. *katoptron* fish were trained to recognize food (∂) associated with high intensity red light (100%, 2 mW/mm^2^, 630 nm). (B) A school of 8 *A*. *katoptron* fish were swimming within the cave area during high intensity (100%, 480 nm) blue light most likely resembling day light conditions and strong activation of the retina. (C) Wavelength dependent feeding behavior of *A*. *katoptron* at high intensity (100%) was measured at 460 nm, 480 nm, 530 nm and 630 nm light beams, at the area where food was normally supplied.
(TIF)

S1 Video Conditioned feeding behavior of *Anomalops katoptron* at high intensity red light. A representative video of behavioral food conditioning experiment from a school of 8 *A*. *katoptron* fish which were trained to recognize food delivery associated with high intensity red light (100% at 630 nm, 2 mW/mm^2^, conditioned stimulus) on the top, left side of the aquarium during feeding to illuminate the feeding area for the experimenter.
(MP4)

S2 Video Conditioned feeding behavior of *Anomalops katoptron* at low intensity blue light. A representative video of behavioral food conditioning at low intensity blue light (10% at 480 nm) attracted the fish to the feeding area.
(MP4)

S3 Video Conditioned feeding behavior of *Anomalops katoptron* at low intensity red light. A representative video of behavioral food conditioning at low intensity red light (10% at 630 nm) did not attract the fish to the feeding area.
(MP4)

S4 Video Conditioned feeding behavior of *Anomalops katoptron* at high intensity blue light. A representative video of behavioral food conditioning at high intensity blue light (100% at 480 nm) did not attract the fish to the feeding area.
(MP4)
