# Supplementary figures and images for: Visual tuning in the flashlight fish Anomalops katoptron to detect blue, bioluminescent light
Source: PLoS One. 2018 Jul 11;13(7):e0198765. doi: 10.1371/journal.pone.0198765 (PMC6040694; doi:10.1371/journal.pone.0198765)

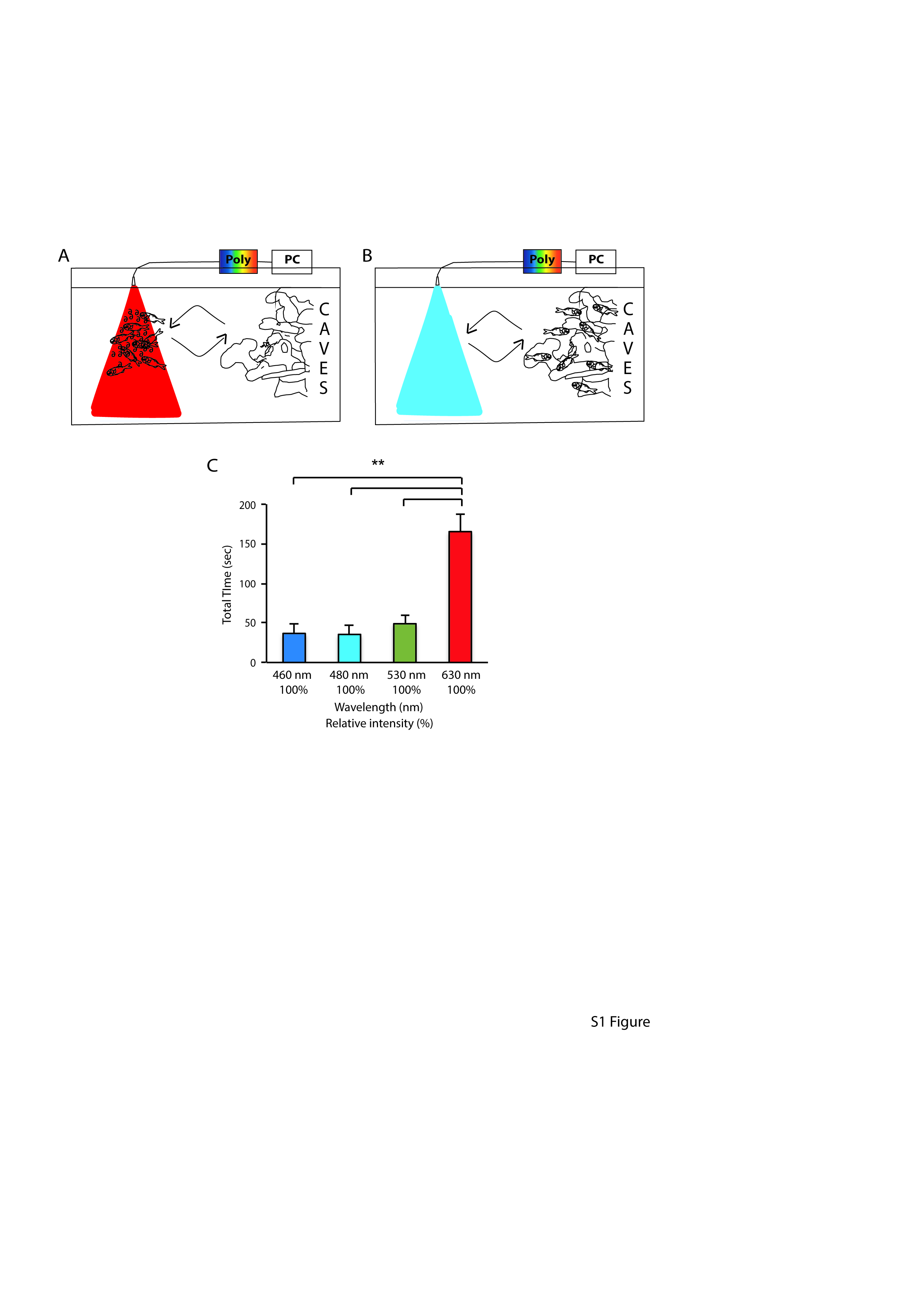

Supplement: S1 Fig — Schematic representation of the behavioral food conditioning experiment (polychromatic light source, Poly). (A) A school of 8 A. katoptron fish were trained to recognize food (∂) associated with high intensity red light (100%, 2 mW/mm2, 630 nm). (B) A school of 8 A. katoptron fish were swimming within the cave area during high intensity (100%, 480 nm) blue light most likely resembling day light conditions and strong activation of the retina. (C) Wavelength dependent feeding behavior of A. katoptron at high intensity (100%) was measured at 460 nm, 480 nm, 530 nm and 630 nm light beams, at the area where food was normally supplied. (TIF) [file pone.0198765.s001.tif]
